# Harm reduction approach in Egypt: the insight of injecting drug users

**DOI:** 10.1186/1477-7517-10-17

**Published:** 2013-10-01

**Authors:** Doaa Oraby

**Affiliations:** 1 Independent consultant, Cairo, Egypt

**Keywords:** AIDS, IDUs, Harm reduction, Egypt

## Abstract

**Background:**

Egypt has low HIV prevalence (below 0∙02%) among the general population, mostly attributed to the conservative culture. The 2010 second round biological/behavioral surveillance survey (Bio-BSS) conducted in some governorates revealed concentrated epidemic among male injecting drug users (IDUs).

**Methods:**

The current study aimed at exploring the perspective of IDUs regarding the HIV preventive efforts targeting them to provide relevant evidence based policy recommendations. The study included desk review, in-depth interviews with service providers and focus group discussions with IDUs of both sexes.

**Results:**

The study described the current harm reduction interventions in Egypt and highlighted the insights of active IDUs and service providers interacting with them as regards their ability to address their needs and what they miss in these interventions and how IDUs perceive these interventions.

**Conclusion:**

The epidemiological reality of HIV infection in Egypt favors prioritizing efforts to the high risk groups rather than the general population. Hence, harm reduction should be at the core of interventions targeting HIV. The current study revealed that there is still a long way to go to enhance the role of these interventions in influencing a significant behavior change among target group.

## Background

HIV has been documented among injecting drug users (IDUs) in at least half of the Middle East North Africa (MENA) countries [1] and injecting drug use has been documented as the dominant transmission mode in the Islamic Republic of Iran (more than 67% of cases) [2] and Libya (as much as 90%) [3]. HIV prevalence among IDUs in MENA countries is in the low to intermediate range compared to other countries around the world [4]. MENA is flooded with inexpensive drugs due to record levels of heroin production in Afghanistan [5].

IDUs face the risks of HIV through sharing of contaminated needles and other drug paraphernalia, and engaging in unprotected sex sometimes occurring under the effect of or in exchange for drugs. Additionally, because of their legal status, IDUs are frequently put in prison, where clean needles are harder to find, thereby raising the threats. Several studies have documented relatively high levels of injection equipment reuse in MENA, such as the reuse of needles or syringes during last injection, last month, last six months, last year, or during lifetime. Levels of hepatitis C virus (HCV), a marker of using non sterile injecting equipment, are in the intermediate to high range, also confirming the potential for further HIV spread [6]. Furthermore, various studies indicated that the majority of IDUs in MENA are sexually active and engage in risky sexual behavior [7]. Once HIV enters a community of IDUs, the infection can rapidly spread through sexual relations to the rest of the population. Being married (currently or at some point) was reported by 55.5% of IDUs in Egypt [8] and, multiple sexual partnerships were also reported by 73% of IDUs in Egypt [9]. Reported high levels of sexual risk behavior, indicate substantial overlap of risks among the three priority groups of IDUs, men who have sex with men (MSM), and female sex workers (FSWs). This IDU risk context suggests the possibility of further concentrated HIV epidemics among IDUs in MENA over the next decade [6].

HIV spread is not a question of law enforcement to prevent risky behavior; repressive measures will only complicate prevention efforts and push risky behavior further underground, making it even more difficult to reach these groups with programs. This would not change the vulnerability settings but would deprive MENA governments and their partners from the ability to prevent the epidemic and administer prevention interventions as needed [6]. With the emergence of IDU as an important driver of the epidemic in MENA, and with the wealth of evidence accumulated around the world regarding the effectiveness of harm reduction interventions in preventing, slowing, or even reversing HIV epidemics among IDUs [10], harm reduction is increasingly appropriate in MENA. MENA countries are at different stages of introducing the different components of the harm reduction package. The Islamic Republic of Iran is a model country in its response to HIV among IDUs, with a rapidly scaled-up plan to make available needles and syringes, opioid substitution therapy (OST), HIV testing and counseling, and sexually transmitted infection (STI) services. Once stabilized on OST, eligible HIV-positive IDUs are provided with antiretroviral therapy (ART) [6].

Egypt is a lower middle-income country situated at the heart of the Middle East and the north-east corner of Africa. Egypt has low HIV prevalence (below 0∙02%) among the general population [11] mostly attributed to the conservative culture. However, the 2010 second round biological/behavioral surveillance survey (Bio-BSS) conducted in some governorates revealed concentrated epidemic among male IDUs [12]. Transmission through IDU represented around 5∙1% of reported cases in 2010 [13]. Drug use is illegal in Egypt; the act is punishable if the person is caught using drugs at time of arrest. If a person reports that he is a drug user to health officials he/ she is treated as a patient and is referred to rehabilitation centers. The estimated number of IDUs in Egypt range from 57,000- 120,000 [14].

According to the United Nations Office on Drugs and Crime, drug abuse in Egypt is mainly a male problem between 20 and 30 years of age although female abusers are increasing and the main drugs of abuse in Egypt are Bango (cannabis herb) and hashish (Cannabis) [15].

Through sharing injection equipment, IDUs are also at particular risk for acquiring hepatitis C virus (HCV) [16]. In Egypt, HCV is one of the major health threats and leading causes of death and the overall prevalence positive for antibody to HCV was 14.7% among representative sample of both urban and rural populations in the entire country [17].

The current study aimed at exploring the perspective of IDUs regarding the HIV preventive efforts targeting them to provide relevant evidence based policy recommendations.

## Methods

The study included desk review of published and unpublished reports, in-depth interviews (IDIs) with key informants and focus group discussions (FGDs) with IDUs of both sexes who utilized the services of harm reduction interventions. The IDIs and FGDs were structured around the themes of current HIV interventions targeting IDUs, their ability to address their needs and what they miss in these interventions and how IDUs perceive these interventions.

Key informants included 20 service providers affiliated to projects targeting IDUs and have been working in that field for at least one year at the time of the study. Five FGDs were conducted with 60 active IDUs who utilized the services of these interventions throughout the period March- May 2011 to gain their insight of these interventions. These active IDUs were approached at exit from the intervention sites where the study was explained to them and if they were interested they were asked to attend FGD meeting within one week at a non- governmental organization (NGOs) premises where discussions were conducted in a quiet confidential environment. The participants of FGDs were offered money equivalent to $5 to compensate for transportation costs. An informed oral consent was obtained from all research participants after explaining the purpose of the study, assuring voluntary participation in addition to asking permission to record the interviews. FGDs and IDIs were transcribed verbatim and transcripts were checked against the audio recordings for accuracy. Patterns in and across the discussions were coded thematically. Data saturation was reached with the data presented here. Analysis was done based on a grounded-theory. Collected data and transcripts did not have identifiers and were stored in a secure place with access only available to research team.

## Results and discussion

Desk review and analysis of IDIs revealed that HIV prevention efforts in Egypt encompass public interventions for general population and recently adopted targeted interventions for high risk groups including IDUs. The public interventions mainly focused on awareness raising sessions targeting youth in schools, universities and youth centers all over Egypt, in addition to commemorating events as World AIDS Day Celebration. These public interventions are mainly implemented by governmental agency-Egypt National AIDS Program- in collaboration with international organizations and NGOs active in the field of HIV. Harm reduction interventions targeting IDUs were established in Egypt in 2008; they were funded by international organizations and implemented by NGOs with active linkages to high risk groups including IDUs, MSM and FSWs. When these interventions were conceptualized, IDUs were considered a safe entry point for the other high risk groups being the least stigmatized, former addicts were appointed as outreach workers being most familiar with congregations of IDUs and how to establish rapport with them and NGOs were selected as the implementing partner to avoid cultural sensitivities in explicit outreach efforts for stigmatized populations. The current interventions have limited outlets and are concentrated in Cairo and Alexandria mainly. The delivered services encompass outreaching IDUs by the former ones working as outreach staff, briefing them on the services delivered at the intervention sites and escorting or referring them to the service delivery sites. Harm reduction intervention model implemented in Egypt include the following services which are offered anonymously and free of charge; peer education on safe sex and safe injection, HIV voluntary counseling and testing using rapid test kits for fast delivery of results, medical services for those in need with special focus on management of sexually transmitted infections. In addition, the services include distribution of behavior change communication booklets and brochures, needles and condoms also free of charge. Active IDUs at FGDs stated that owing to Egypt conservative culture and the sensitivity of the drug use issue they feared disclosing their practice and asking for advice at the health facilities they were familiar with.

*“We had no place to go to and say we are addicts When we are ill, we do not tell the doctors we are addicts…but I feel they know from marks on my arm and treat us with fear”* Male IDU.

Though most supporting evidence of harm reduction interventions stem from studies in resource-rich countries or countries culturally different from Egypt conservative culture, yet harm reduction is supported by the two key tenets of Islam, Egypt prevalent religion, doing no harm to oneself or others and the worst harm is eliminated by a lesser harm [18].

*“Some clients suspect the intentions of the services wondering why they would provide such services free of charge”* Female outreach worker.

Competent outreach workers can bring prevention services to the hard-to-reach IDUs (who can serve as a safe entry point to FSWs and MSM who inject drugs)and help establish trust between them and other health and social services [19]. Participants of FGDs valued outreach workers as the established rapport and trust with outreach workers –who are former IDUs- made them feel cared for, instead of fearful or threatened. Outreach workers-as revealed by IDUs- use words and examples that they could understand, do not speak in a judgmental manner and draw from their experiences to help active IDUs modify unsafe behaviors.

*“I felt I am at home speaking to my friends”* Female IDU.

Low levels of needle-syringe programs (NSP) coverage may be sufficient to sustain an effective response to HIV prevention if clean injecting equipment is also available from other sources, such as pharmacies [20]. Affordable sterile syringes are available at pharmacies in Egypt. When inquired about delivered services through targeted interventions, both active IDUs and service providers clarified that although syringes are distributed to them without exchange and free of charge yet their uptake is limited. IDUs added that they fear being detained as carrying injecting equipment, particularly if drug contaminated is a sufficient excuse for police arrest.

*“If I am caught with a syringe I will be imprisoned.”* Male IDU.

Methadone is not distributed through current harm reduction interventions implemented in Egypt. IDIs with service providers revealed that opioid substitution is still an issue of debate among health professionals who believe that opioid substitution programs are not appropriate, feasible, or affordable for Egypt and can be diverted to illicit markets. Alternatively, they encourage immediate abstinence from drug use, rather than the gradual process that methadone substitution therapy entails; addicts are often given sedatives and painkillers to cope with withdrawal symptoms. However, research on the utility of drug dependence treatment as an HIV-prevention strategy has focused primarily on methadone maintenance treatment [21]. In Egypt, narcotic anonymous (NA) is an important treatment option for opioid-dependent people who cannot gain access to methadone. Recovery involves daily prayer and meditation to maintain conscious contact with God and to seek his will and power [22], which conforms with the deeply rooted Egypt religious culture in IDUs and their acquaintances. Christians recapture its Christian roots and Muslims observe shared connection between Islam and certain aspects of the 12 Steps. However, evidence of the effectiveness of NA in HIV prevention is limited. Key findings of Fiorentine longitudinal study [23] include that weekly or more frequent 12-step participation may be an effective step in maintaining relatively long-term abstinence.

When asked about what they miss in the harm reduction interventions, IDUs at FGDs stated that that the most prevalent and pressing health issue among themselves is HCV, and neither HCV diagnosis nor management is provided through current interventions.

*“Services at the place are free and we pay a lot of money for HCV treatment…can they be included?”* Male IDU.

The similar modes of transmission of HIV and HCV present a unique opportunity to provide prevention services at a single client visit to the harm reduction service delivery points. Addressing the HCV needs of IDUs will increase their acceptability and buy in of HIV prevention services delivered at the same place and will probably improve their response to risk reduction interventions.

IDUs at FGDs also added that the currently delivered services do not support those who wish to quit addiction but are economically hindered. Some of them viewed this shortcoming as *“a westernized agenda for legitimization of risky behaviors”.* Interviewed service providers added that when faced with such situation, they guide those who wish to quit to NA meetings. Most of the participants of FGDs are jobless and hence they recommended linking the harm reduction interventions to microfinance project or inclusion of vocational training and computer courses in the package of delivered services thus helping them to generate income and may motivate them to quit addiction. For most interviewed IDUs, poor and unemployed, getting high is their only escape from the hardships they face daily. Hence, assisting them in overcoming their daily challenge through provision of or linking harm reduction interventions to income generating activities will increase their acceptance and buy in of the delivered services.

*“Youth escape to drugs because of unemployment.”* Female IDU.

Most of the IDUs at FGDs were married mostly to addicts like themselves and have children. Some IDUs at FGDs revealed that under the effect of drugs, safe sex can never be guaranteed.

Interventions to reduce the sexual risk behaviors of IDUs, including condom provision and improved access to sexual health services, have a modest impact on HIV transmission than those that reduce injecting risk behaviors [24]. The 2010 Bio-BSS revealed that despite the numerous prevention interventions targeting IDUs, unprotected sex is prevalent and condom is inadequately used [12].

*“When I am high, I do not know with whom I am sleeping (having sexual relation) so do not ask me about condom”* Male IDU.

## Conclusion

The epidemiological reality of HIV infection in Egypt favors prioritizing efforts to the high risk groups rather than the general population. Hence, harm reduction should be at the core of interventions targeting HIV, being the most direct and effective strategy to avert the tide of HIV through addressing the root causes of risks and vulnerabilities. However, the current study revealed that there is still a long way to go to enhance the role of these interventions in influencing a significant behavior change among target group.

A structural weakness of the HIV response in MENA is the insufficient contribution of people living with HIV groups in the formulation, planning, and implementation of the response. Listening to IDUs and addressing their concerns and recommendations will scale up harm reduction interventions in Egypt and eventually enhance and sustain HIV prevention efforts. The below recommendations are meant to initiate a dialogue among stakeholders in Egypt and MENA region.

• Strengthening NGOs given the epidemiological reality of HIV transmission being concentrated among largely hidden and stigmatized populations.

• Expanding coverage and intensifying implementation of the harm reduction interventions.

• Conducting research to assess the role of NA in empowering IDUs and enabling them to adopt safe behaviors or quit drug addiction.

## Abbreviations

Bio-BSS: Biological/Behavioral surveillance survey; FGDs: Focus group discussions; HCV: Hepatitis C virus; IDIs: In-depth interviews; IDUs: Injecting drug users.

## Competing interests

The author declares that she has no competing interests.
